# Optimising opportunities for self-collection to improve cervical screening access for people with physical disability in Australia: a qualitative study of service provider perspectives

**DOI:** 10.1186/s12889-026-28364-3

**Published:** 2026-07-10

**Authors:** Kate Flynn, Tessa Saunders, Amelia Hyatt, Claire Bavor, Julia ML Brotherton, Deborah Bateson, Nicola S Creagh, Karleen Plunkett, Louise Bannister, Madeleine Clarke, Natalie Taylor, Paula Jops, Allison Cummins, Angela Kelly-Hanku, Megan Smith, Claire Nightingale

**Affiliations:** 1 Evaluation and Implementation Science Unit, Melbourne School of Population and Global Health, The University of Melbourne, Melbourne, Victoria Australia; 2 Australian Centre for the Prevention of Cervical Cancer, Melbourne, Australia; 3 School of Nursing and Midwifery, College of Health Medicine and Wellbeing, University of Newcastle, Newcastle, NSW Australia; 4 Cancer Elimination Collaboration, Sydney School of Public Health, Faculty of Medicine and Health, The University of Sydney, Sydney, NSW Australia; 5 Implementation to Impact (i2i), School of Population Health, UNSW Sydney, Sydney, Australia; 6 Kirby Institute, UNSW, Sydney, Australia; 7 Specialty of Obstetrics, Gynaecology and Neonatology, Faculty of Medicine and Health, Sydney Medical School, at the University of Sydney, Sydney, Australia; 8 Independent consultant with lived experience of disability, Victoria, Australia; 9 Independent consultant with lived experience of disability, Canberra, Australia; 10 School of Health Sciences, The University of Sydney, Sydney, Australia

**Keywords:** Cervical screening, Self-collection, Physical disability, Access to healthcare, Preventative health, Service providers, Nurses, Scope of practice, Policy

## Abstract

**Objective:**

People with physical disability experience systemic and societal barriers to accessing cervical screening. Service providers play an important role in supporting access to cervical screening for this population. In 2017 Australia’s National Cervical Screening Program introduced the choice to self-collect using a vaginal swab, with universal access available from July 2022. This study aimed to understand service providers’ perspectives on the opportunities self-collection provides to improve access to cervical screening for people with physical disability.

**Methods:**

Semi-structured interviews were conducted online between June and August 2023 with 22 clinical and non-clinical service providers from all States and Territories except the Northern Territory. The Updated Consolidated Framework for Implementation Research informed the analysis.

**Results:**

All participants believed that self-collection was a highly acceptable option, both for people with physical disability and service providers, due to its potential to reduce anxiety and discomfort for screening participants, and the opportunities it provides to increase access to screening in non-clinical settings, including at home and in supported accommodation. To date, few services providing disability support had integrated self-collection into their practice. Key barriers to offering self-collection included confusion regarding whether cervical screening was within all provider’s scope of practice, a lack of communication between the health system and the disability sector and an absence of education and resources for providers and people with disability. Attitudes toward people with disability held by healthcare providers, such as general practitioners and other health and wellbeing services, and the quality of relationships between services were seen as either barriers or enablers depending on whether these were positive or not. Several providers expressed concern that the swab may be difficult for some people with physical and sensory disability to use, with adaptations recommended.

**Conclusion:**

Self-collection offers opportunities to improve access to cervical screening for people with physical disability, if supported by providers who have the required knowledge and skills.

Implementation strategies to support uptake of self-collection for people with physical disability are required at the individual, device, service, and system level.

**Supplementary Information:**

The online version contains supplementary material available at https://doi.org/10.1186/s12889-026-28364-3.

## Introduction

In 2020 the World Health Organization launched a global strategy to eliminate cervical cancer [1], however, access to cervical screening remains inequitable both within and between countries [2, 3].

Australia published a national cervical cancer elimination strategy (national strategy) in 2023 with a key focus on equity, particularly for under-screened populations [3]. People with disability are explicitly identified as a priority within this national strategy [3]. Australia has had an organised screening program since 1991, with a five-year coverage rate of 73% of the eligible population in 2024 [4]. Since December 2017, Australia’s National Cervical Screening Program (NCSP) transitioned to human papillomavirus (HPV) based screening, and recommends all women and people with a cervix [5] aged 25–74 who have ever been sexually active screen every five years [6]. Since July 2022, Australia’s NCSP has offered two screening options: clinician-collection with a speculum, and self-collection from the vagina with a soft, thin swab [7]. In Australia, self-collection must be ordered and overseen by a healthcare professional and, to obtain reimbursement under the universal insurance scheme, they must also have a Medicare provider number. This largely limits this role to general practitioners, nurse practitioners and specialists [8]. Nurses and midwives in Australia are able to undertake cervical screening provider training, however their capacity to practice autonomously varies.

There is no national screening participation data for people with disability in Australia; however, evidence from other settings suggests screening rates are lower among this population than the general population [9–17]. Approximately one in five Australians live with disability, most commonly physical disability, and prevalence increases across adulthood, encompassing a substantial proportion of the screen-eligible population [18]. Significant barriers to cervical screening participation for people with physical disability occur at an interpersonal, systemic and societal level. These include: lack of appropriate equipment, including hoists and adjustable beds; healthcare provider and carer beliefs that people with disability do not require cervical screening due to the assumed absence of sexual activity; short appointment times; and a lack of accessible informational resources [15, 16, 19].

Australian and international evidence shows self-collection is highly acceptable for many people who experience barriers to accessing the clinician-collected test [20–24] and may offer new opportunities to facilitate cervical screening for people with disability [25]. As one strategy to increase access to screening, Australia’s national strategy identifies nurses, midwives and Aboriginal Health Workers and Practitioners without provider numbers as potentially having a greater role in facilitating access [3].

However, previous research has demonstrated gaps in professional development for nurses in how to best support people with physical disability [26], and the role of disability support services has not yet been explored.

This study aimed to explore service provider’s perspectives on how self-collection could increase cervical screening participation for people with physical disability, and what is needed to support this.

### Terminology and definition of physical disability

Following discussion with community advisors with lived experience of disability, we adopted the following definition of physical disability, created by people with disability:

*“A physical condition that affects a person’s mobility*,* physical capacity*,* stamina*,* or dexterity.” *[27] *This definition also captures some chronic illnesses.*

We acknowledge that women, non-binary and gender diverse people with a cervix who have a physical disability come from different cultures and backgrounds, have diverse sexual identities, and are not a homogenous group. The words “people with physical disability” will be used throughout this paper to denote this diverse target cohort, and “people with disability” when speaking more broadly.

## Methods

### Study design

The study was designed in consultation with a Community and Consumer Advisory Panel which includes women with physical disabilities. We employed online qualitative semi-structured interviews to gain in-depth insights into the perspectives of service providers working with people with physical disability. This study is part of a larger program of work investigating methods to improve equity of access to cervical screening for Australian populations identified in our National Strategy, including self-collection.

### Participants and recruitment

Service providers who deliver health and/or care services to people with physical disability in Australia were recruited purposively [28] through professional contacts and peak disability organisations via email, newsletters and social media. Snowball sampling [29] was employed with participants recommending other eligible services to contact. Participants were not required to be currently involved in providing or promoting cervical screening, as an aim of this study was to explore whether self-collection now offers opportunities for providers to be involved in different ways that were not possible with clinician-collection. Participants received a $100 gift card if they participated outside of their salaried work. Participants received a plain language statement detailing study purpose, and a consent form via email. No relationships were established with participants prior to the commencement of this study.

### Data collection

Interviews were conducted by authors KF and TS, who are both cisgender, non-disabled women. KF is a registered nurse with experience in health interviewing and was a Master of Public Health student for the duration of this study. TS is a post-doctoral research fellow with extensive experience conducting qualitative interviews. An interview guide was developed for this study and informed by the Updated Consolidated Framework for Implementation Research (CFIR 2.0) [30]. Interviews covered service provider’s perspectives and experiences of offering and/or supporting access to cervical screening for people with physical disability, prior knowledge of self-collection as an option for cervical screening, whether self-collection offers new opportunities within their roles, and what would be needed to support this. The interview guide was tested for face validity and flow by members of the research team with relevant experience. Prior to commencing the interview, information about the research project and the roles and relevant experience of the research team was explained to participants, along with information about recent changes to the NCSP and the option for self-collection. Interviews were ceased when no new topics and information were emerging, and data saturation was reached. A copy of the interview guide is available as supplementary material. This study followed the COREQ guidelines [31].

### Data analysis

Interviews were audio recorded and transcribed verbatim. De-identified transcripts were sent to participants for review with suggested changes considered and made. Transcripts were then coded deductively in NVivo 13 by a single author (KF) and discussed with authors TS and AH for rigour. A staged process was utilised to develop the coding framework, beginning with Braun and Clarke’s thematic analysis [32] to map codes, categories and themes to the study research questions. Findings were then integrated with the CFIR 2.0 Framework in the second stage. CFIR 2.0 is a conceptual framework comprising five higher level ‘domains’ and corresponding ‘constructs’ designed to evaluate implementation of an innovation or intervention at an early stage to identify areas for improvement (Table 2) [30]. The final coding framework therefore comprised a combination of thematic findings organised within relevant CFIR 2.0 domains to aid systematic understanding and interpretation of data regarding the extent to which cervical screening provision or promotion is currently occurring within services, and what people perceived they needed to support a greater role.

## Results

Twenty-two service providers, from 15 different organisations, were interviewed via Zoom between June and August 2023. A total of 18 interviews were conducted (17 individual and 1 group). Interview length ranged from 24 to 72 min (mean: 53 min). Most (14) participants were in non-clinical roles (Table 1). Participants were recruited from seven of the eight Australian States and Territories.

**Table 1:** Participant characteristics

Category	Individual participants (22) *n* (%)	Organisations (15) *n* (%)
**Role type**		
^Clinical (non-cervical screening provider, nurse, midwife, physician who provide healthcare to people with disability)	8 (36%)	
Non-clinical (disability support worker and house supervisor, educator, counsellor, practice manager, all providing disability support services)	14 (64%)	
**Service type individuals work within**		
Sexual and reproductive health, medical, general practice and home visiting nursing service	6 (27%)	6 (40%)
Disability advocacy and support services (including supported accommodation)	11 (50%)	4 (27%)
State funded health services (including community health services)	5 (23%)	5 (33%)
**Interview type**		
Individual interview	17 (78%)	17 (94%)
Group interview	5 (22%)	1 (6%)
**Service geographical location**		
Victoria	6 (27%)	4 (26%)
New South Wales	4 (18%)	4 (26%)
Other* (Queensland, Western Australia, South Australia, Tasmania, Australian Capital Territory)	12 (54%)	7 (46%%)
**Gender**	**Individuals (** ***n*** ** and %)**	
Woman	15 (68%)	
Man	1 (5%)	
Non-binary and other	0 (0%)	
Missing^a^	6 (27%)	

^of these, 4 were cervical screening providers

*Grouped to maintain confidentiality, as all were *n<*3

^a^Gender data was not initially collected and is missing for 6 participants

### Interview findings

We identified six themes which aligned with the CFIR 2.0 domains and constructs (Table 2); (1) Opportunities to increase cervical screening through use of and access to self-collection, (2) Scope of practice ambiguity, (3) Disconnect between the public health system and the disability sector, (4) Attitudes towards people with disability, (5) Cross service relationships, and (6) Access to training and accessible information and services (Table 2).

**Table 2:** CFIR 2.0 domains, constructs and corresponding themes

Finding	CFIR 2.0 Domain + description	CFIR 2.0 Construct	Example in this study
Opportunities to increase cervical screening through use of and access to self-collection	Implementation Process *Activities to implement the innovation in the inner setting*	Adapting Relative priority	• Recognition of key role for community-based nurses and midwives to offer self-collection in the home and in supported accommodation
	Inner Setting *Setting where the innovation is being/could be implemented (the healthcare and/or disability support service)*	Tension for Change	• Healthcare and disability service providers are eager to improve client access to cervical screening through the opportunities self-collection offers
	Innovation *Evidence-based product/practice being implemented i.e.*,* self-collection*	Innovation Design Innovation Complexity	• Adaptions to self-collection device/delivery recommended to improve usability
Scope of practice ambiguity	Outer setting *Settings/factors occurring outside of service (inner setting) which impacts on the services process of implementation*	Policies and Laws	• Clarification of national screening policy required to support identified roles for community nurses to offer self-collection in client’s homes
	Individuals *Roles and characteristics of people delivering or receiving the innovation*	Opportunity	• Opportunity for nurses’ and midwives’ scope of practice to be expanded with flexible self-collection delivery and funding models
	Inner Setting	Compatibility	• Disability support service policies prohibiting the insertion of items (e.g., vaginal swab) into clients
Disconnect between the public health system and disability sector	Outer Setting	Policies and Laws Partnerships and Connections	• Confusion over which screening support services are covered by the health system and disability sector
Attitudes towards people with disability	Inner Setting Outer Setting	Relational Connections Local Attitudes	• Misconceptions held by other disability and health service providers (general practitioners and nurses), carers and family that people with disability are never sexually active and do not need cervical screening
Cross-service relationships	Outer setting	Partnerships and connections	• Referral pathways in place from GPs to nurses to provide cervical screening
Access to training and accessible information and services	Inner Setting	Access to Knowledge and Information	• Lack of trauma-informed training for healthcare providers as a barrier to supporting screening access • A need for accessible resources in different formats

Overall, study participants perceived that participation in cervical screening was either rare or did not occur for people with physical disability. Further, there was low awareness of where people with physical disability could access cervical screening. This perspective was consistent across metro and regional areas, with access to cervical screening for people with physical disability perceived to be poor irrespective of geographic area. However, access to transportation to attend cervical screening was perceived to be more limited in regional and rural areas for this population.

Prior awareness of the National Cervical Screening Program and of the self-collection option varied widely among participants, with those working in clinical roles more likely to state they had prior knowledge of cervical screening options available within the national program. All participants identified ways they could support or provide self-collection in their current roles to overcome low rates of participation but shared a need for clarified policies and scope of practice to support this. 

### Opportunities to increase cervical screening through use of and access to self-collection (implementation process, inner setting, innovation)

Regarding the relative advantage of the ‘innovation’ itself, self-collection was seen as an improvement on current cervical screening practice. All participants saw an opportunity to increase access and improve the experience of screening by bringing self-collection into their client’s individual homes and supported accommodation. Provided the appropriate support was available, all participants viewed offering or supporting self-collection in these locations as compatible with current workflow.

Some clinical providers spoke of discussions within their services to start offering self-collection for clients in their home; however, there were some challenges with the implementation process and these had not progressed due to (1) competing demands of health services, and (2) uncertainty over how to build partnerships with disability service providers, reflecting barriers within the CFIR 2.0 constructs of ‘relative priority’ and ‘partnerships and connections’. A smaller number of nurses (3) working in community outreach reported already offering assisted self-collection to their clients, which is defined as a healthcare provider assisting someone to collect their own vaginal sample if the patient does not feel able or confident to do so themselves [8].

Many participants reflected that nurses undertaking home-visiting services could assist clients to self-collect, if it was introduced into the services they offer with appropriate training, funding, workplace policies and resources, however, to date, only a smaller number of nurses had been doing this.

*“We would work with them to get the kits home*,* then we would help them*,* we could help them to actually take their own sample.”* Participant 6 (Nurse)

Despite self-identifying as offering cervical screening services for people with disability, several clinical service providers reported not having the necessary equipment such as hoists and adjustable beds, reflecting a gap in ‘physical infrastructure’ within the inner setting domain. Offering self-collection in flexible locations such as supported accommodation was seen as an advantageous way to address the lack of appropriate equipment to support cervical screening for people with physical disability:

*“So in clinic settings*,* we’ve got beds that are able to be moved up and down*,* laid back*,* sat up… as women’s health nurses we’re very flexible*,*… if that disability meant that they were unable to attend that service*,* because we may not have a hoist or something … then we would offer it at home because it’s often less traumatic for them …”* Participant 10 (Nurse)

Participants perceived self-collection as being highly acceptable with many advantages compared to current practice, including that the swab was “less invasive” and “offered more control” than the clinician-collected test for people with physical disability, and particularly relevant for people whose limited mobility impacted the insertion of a speculum. This also reflects greater adaptability of self-collection, which is a key construct within CFIR 2.0 that allows an innovation to be tailored to local context and needs [30]. Nurse cervical screening providers suggested that stability supports, such as handrails in clinic bathrooms, enabled some people with physical disability to take their own sample independently, but these supports were not always available.

Several participants identified physical barriers to the use of the self-collection swab (the “innovation”) for people whose limited dexterity or mobility would prevent them from using the device in its current form, notably that the swab and encasing tube are very thin and require fine motor skills to utilise. An adapter tool, or a bump for people with low vision, were recommended as necessary to allow people with physical and sensory disability to be able to use the swab without assistance.

*“I wish… there would be a swab that’s really going to be developed someday*,* where they [people with physical disability] would actually be able to grip it*,* where it’s designed to actually help them grip it safely. I wish there is that tool to help so that they could really do it [cervical screening] by themselves and you’re just there trying to support them*,* making sure they’re safe. I think it would be revolutionary.”* Participant 12 (Nurse)

### Scope of practice ambiguity (outer setting, individuals, inner setting)

Most barriers described by service providers occurred at both an external level “outer setting” and within their organisation’s “inner setting” (Table 2). Uncertainty about national and organisational policies left many participants confused about their scope of practice and the degree to which they could implement self-collection within their service. These participants identified a need for further clarification of roles, processes and policies for different types of providers to support clients with self-collection. Clinical providers (both cervical screening and non-cervical screening providers) frequently queried whether training was available for registered nurses regarding ordering and providing self-collection only (not clinician-collection), as this would allow them to reach clients outside the clinic who face barriers in accessing local health services. Participants recognised a change to current policy on who can order self-collection was needed to support these opportunities.

Exploration of funding models to support nurses to travel to individual homes and supported accommodation was highlighted by participants.

*“I think sometimes we just get caught up in too much red tape. Nurses should be able to provide a self-collected kit at home*,* going to where the people are that aren’t screening*,* or where there’s nobody else to do it*,* nurses have historically been great at doing that sort of work. If the health service will provide some funding to do that*,* then we should be doing it.”* Participant 10 (Nurse)

Many supported accommodation services for people with disability have policies in place to protect clients which prohibit the insertion of tampons for example, and disability support workers shared discomfort with the idea of assisting their clients to self-collect. A preferred option among these participants was to facilitate a community nurse to visit the supported accommodation to offer self-collection.

*“That’d be something maybe we could have nurses come out to do at the house*,* we would have to organise for the nurses to come out to do that*,* which we do anyway for other needs of our clients.”* Participant 2 (House Supervisor)

Whilst some services did have policies which allowed non-clinical staff to support clients with insertion of suppositories or performing enemas, additional training and adaptations to organisational policy would be required to facilitate access to self-collection:

*“And I think for some of our clients who perhaps have some*,* perhaps physical challenges to doing it [self-collection]*,* I can imagine that the quality and safeguarding team would get lots of questions from frontline staff in terms of wanting to know what’s appropriate in terms of how much they can assist with that process.”* Participant 15 (Manager)

### Disconnect between the public health system and disability sector (outer setting)

A further “outer setting” barrier to services supporting cervical screening was the lack of communication between the public health system and disability sector, particularly Australia’s National Disability Insurance Scheme (NDIS). CFIR 2.0 acknowledges the importance of ‘policies and laws’ which support the implementation and delivery of an innovation. Non-clinical providers described confusion over NDIS coverage vs public health system coverage to assist clients to access cervical screening supports, such as financial responsibility for transportation to cervical screening appointments. One participant discussed how a lack of coordination between the NDIS and the health system has resulted in the sexual and reproductive health needs of people with disability being missed:

*“One of our concerns has been that a lot of reproductive and sexual health issues are falling in that gap*,* where I mean cervical screening test*,* that’s health*,* but if a person needs support to get to the appointment*,* that’s NDIS. And you know*,* obviously they need to work together*,* and I would say that they’re generally not working very well together.”* Participant 8 (Health Promotion Manager)

Strengthening relationships between the public health system and the disability sector was discussed as a requirement to support streamlined access to safe and appropriate cervical screening services for people with physical disability.

### Attitudes towards people with disability (inner setting, outer setting)

Many participants spoke of misguided attitudes held by healthcare providers (general practitioners and nurses), carers and families toward people with disability and their sexual health needs as inhibiting access to cervical screening for their clients. This links back to the CFIR 2.0 construct of ‘local attitudes’ which recognises that social and cultural values can impact on implementation. This included beliefs that people with disability are never sexually active, and paternalistic attitudes held by family members. Some participants felt the option for self-collection, on its own, would not be sufficient to address these underlying attitudes.

*“People believe that… people with disabilities aren’t sexual… and therefore*,* they don’t have access to this sort of information*,* they’re not provided education”* Participant 1 (Counsellor)

### Cross-service relationships (outer setting)

Cross-service relationships (or ‘partnerships and connections’) were an enabler to supporting access to cervical screening. For some services this took the form of a local male general practitioner referring their patients with disability to the local nursing service for provision of cervical screening:

*“We work really closely with GPs*,* there’s a lot of male GPs in these areas who don’t screen so we would work with them*,* they refer the patients*,* we do the cervical screening*,* that’s worked really*,* really well.”* Participant 10 (Nurse)

The benefits of working relationships between services were highlighted by all participants, with many disability support providers and coordinators sharing positive experiences of partnering with community nursing services to offer necessary healthcare for their clients.

### Access to training and accessible information and services (inner setting)

Having ‘access to knowledge and information’ through training and guidance are key CFIR 2.0 constructs to support implementation of a new innovation by staff. Disability support workers and coordinators described significant challenges in finding trauma-informed healthcare professionals and services to provide clinical care for their clients, many of whom had histories of trauma or sexual assault. Participants highlighted a clear need for disability-specific trauma-informed training to increase the capability of healthcare professionals to address this gap in service provision:

*“I wish there was a bunch of doctors that were just educated in disabilities that could be for people with disabilities.”* Participant 2 (House Supervisor)

Participants saw access to knowledge and information as both a barrier and enabler when supporting cervical screening access for their clients. Notably, a lack of access to information for staff about self-collection and how to support clients to screen was limiting non-clinical staff’s ability to play a greater role in this area. This was exemplified by the fact that most non-clinical providers were unaware of self-collection prior to their involvement in our interviews:

*“No…I’ve never heard of it [self-collection].”* Participant 13 (House Supervisor)

*“Yeah*,* I think self-collection could be a winner…I didn’t even know about that.”* Participant 9 (House Supervisor)

Participants who had a current role in offering or supporting access to cervical screening for their clients described access to resources in formats such as audio-visual and easy read as important for promoting informed decision-making among their clients. However, many of these providers stated that current cervical screening resources were not accessible or inclusive of people with disability, leaving many services to create their own. These providers highlighted a need for disability inclusive resources to be co-designed and made widely available.

*“Unless it [resource] is specifically made with that disability mindset*,* a lot of it can totally miss the mark.”* Participant 7 (Educator)

## Discussion

Self-collection offers new opportunities to increase participation in Australia’s NCSP, including for people with physical disability. Our study explored some of this potential from the perspective of people who provide healthcare and disability support services to this population. We found high levels of acceptability for self-collection and recognition of opportunities, but a lack of clarity about how these could be realised, and multiple barriers to implementation. The CFIR 2.0 determinants framework aided in the identification and prioritisation of potential solutions, notably the constructs of: policies and laws, partnerships and connections, access to knowledge and information, innovation complexity and innovation design.

In our study, participants reflected on systemic challenges to supporting access to screening, including lack of detail in relevant policies and laws’ and confusion regarding both clinical and non-clinical provider’s scope of practice. This confusion was impeding opportunities for community nurses and midwives to offer self-collection in clients’ homes and supported accommodation. Previous studies have highlighted a clear need for an increased and clarified scope of practice for registered nurses in relation to broader disease prevention and management, and to alleviate the growing burden on general practitioners [33, 34].

Providing self-collection in the home, when supported by healthcare providers with the necessary knowledge and skills, could offer opportunities to improve screening access. This aligns with previous Australian and international findings [21, 35–37]. Flexibility in Australia’s NCSP guidelines allows for self-collection to be undertaken in locations deemed appropriate by the requesting provider, with the option of practitioner-supported collection if requested [8]. NCSP documentation consistently refers to “healthcare providers” but does not specifically define the type of providers this includes. While this definition deliberately allows for flexibility, it has also created ambiguity, which was challenging for all interviewed services. For cervical screening taking place in healthcare settings, clear standards and financial support for health services are needed, to ensure the accessibility of their service. The Royal Australian College of General Practitioners states that health services must have the “physical infrastructure” to ensure accessibility of the service for people with disability [38], however equipment such as hoists and adjustable beds are not specifically mentioned. Further, health services are obligated under the Disability Discrimination Act 1992 [39] and relevant State and Territory legislation to make reasonable adjustments to services. The expansion and enforcement of these standards is particularly pertinent for services which advertise as disability inclusive and accessible.

This study shows the critical need for policy guidelines (CFIR 2.0 ‘policies and laws’) to recognise and unambiguously endorse varying provider types as being appropriate and valuable to reach under-screened populations, within their scope of practice. This is also highlighted in Australia’s national strategy which states that “expanding who can offer these [screening and colposcopy] services, and where and how they are offered” is key to increasing access to these services and thereby address issues of inequity [3]. Self-collection must be offered appropriately and safely, irrespective of provider type, and by a provider who is acceptable to the person having the test.

This study identified that cross-service relationships (CFIR2.0: ‘partnerships and connections’) could both support or hinder healthcare and disability service providers’ ability to support access to cervical screening. The disconnect between the NDIS and public health system was highlighted by providers’ confusion over who takes responsibility for screening support services. This disconnect needs addressing at a national level, as collaboration between sectors is critical for successful implementation of preventative health services for people with physical disability, such as cervical screening. As described in CFIR 2.0, strong ‘partnerships and connections’ including networks for referral, along with clear policies and guidelines, are needed to support implementation [30]. Without clarity on what screening-related services can be provided and by whom, supporting clients to access cervical screening will continue to be difficult.

As outlined in the national strategy, and in broader calls, greater involvement of people with disability in designing models of service delivery is likely to increase their appropriateness and acceptability [3, 40].

Many service providers could play a greater role in raising awareness of cervical screening options for their clients and supporting them to access self-collection through local health services. This requires that disability service providers have access to professional development to support their clients. Disability inclusive resources in a variety of formats are required to overcome the lack of ‘access to knowledge and information’ for providers to better support their clients’ informed decision making.

Further, professional development centred around delivering trauma-informed care is a priority for healthcare providers to ensure they are meeting the needs of people with disability. There was an appetite for disability service providers to work in partnership with nursing services to facilitate access to home-based cervical screening. This could be further explored through pilot projects to assess the feasibility, acceptability and appropriateness for both services and clients.

Service providers perceived that the self-collection device was inaccessible for people with dexterity challenges and/or sensory disability. This finding, which aligns with the construct of ‘innovation complexity’, needs to be confirmed by people with disability themselves. Preferences, such as size of available self-collection swabs in the UK and the United States, have been explored in previous studies, although these were not specific to people with dexterity challenges [41–43]. Adaptations and/or adaptor tools were suggested to improve usability and address the accessibility of the devices available in Australia, to improve ‘innovation design’ factors such as grip. While there is a clear need to prioritise the development of such tools, adaptations to the device itself would pose logistical challenges, such as the extensive swab validation process that pathology centres in Australia are required to undergo [44]. Any adaptations or design changes must be explored in partnership with people with disability.

### Strengths

This study, which was designed in consultation with people with disability, addresses a clear gap in evidence and can be utilised to inform future practice and policy decisions relating to cervical screening for people with disability. The healthcare and disability service providers recruited for this study had a variety of professional backgrounds, which allowed for a diversity of insights to be explored. The use of CFIR 2.0 offered structure for analysis in the implementation context providers work in, with this implementation focus also allowing identification of challenges unique to the disability sector.

### Limitations

The low number of community nurses recruited impacted the depth of understanding of how self-collection could be offered in the home. Further, generalisability of these findings to the clinical workforce is limited due to the higher number of non-clinical providers interviewed for this study. We recommend further research with clinical healthcare providers to further understand how self-collection can be implemented as a screening choice for people with physical disability in these settings. Additionally, while we were able to recruit in most Australian States and Territories, no providers in the Northern Territory responded to recruitment invitations which limits the national generalisability of this study’s findings. We acknowledge that a large proportion of the Northern Territory is considered geographically remote/very remote and has a large population of Aboriginal and Torres Strait Islander people [45]. Therefore, the Northern Territory healthcare and disability workforce may have additional needs that may not be represented by other Australian States and Territories. It was outside the scope of this study to explore perspectives of people with disability on self-collection opportunities: this is currently being undertaken by the research team in a parallel study. This limits our findings to provider perspectives only.

## Conclusion

This study identified opportunities that self-collection offers for healthcare and disability service providers to improve access to cervical screening for people with physical disability. However, these opportunities rely on barriers at the personal, service, and system level being addressed.

Our findings have implications for policymakers and all service providers, demonstrating the need for an expanded scope of practice for nurses and midwives and increased consultation with people with disability about the acceptability and feasibility of screening models and service provision. Further, people with disability need to be included in the design of any initiatives for them, including adaptations to the self-collection swab, informational resources, and new service models.

## Supplementary Information

Supplementary Material 1.

## Data Availability

The datasets generated during the current study are available from the corresponding author on reasonable request and HREC approval conditions.
