# Supplementary material for: Optimising opportunities for self-collection to improve cervical screening access for people with physical disability in Australia: a qualitative study of service provider perspectives
Source: BMC Public Health. 2026 Jul 10;26:3314. doi: 10.1186/s12889-026-28364-3 (PMC13753260; doi:10.1186/s12889-026-28364-3)
Supplement: Supplementary file 1 [file 12889_2026_28364_MOESM1_ESM.docx]

**Interview Guide for Health Services who provide care/services to people from priority population groups**

**Notes for interviewer/s:**

***Introduce project, interviewer/s and run through consent script prior to starting interview.***

***My name is xxx***

***Supporting Choice is*** *a national project that aims to generate evidence about what is needed to implement the choice for self-collection in ways that are equitable for everyone who is eligible for cervical screening in Australia.*

*We are interviewing people who work in health services that provide care specifically for groups of people who are known to experience additional barriers to screening. We want to understand your experiences with supporting your clients to access cervical screening, what you see as the key enablers and barriers, what opportunities self-collection provides, and what else is needed to ensure more people can equitably access screening. We also have a few quick questions about HPV vaccination, which is a key pillar in the global strategy to eliminate cervical cancer.*

***If you are unsure about any of the questions****, feel free to let me know if there is someone else in the service that we could talk to about this later. Please just answer questions that you are able to and can skip those that you are unsure about. I will ask you to provide the name and contact details of anyone else that you think we should talk to.*

***QUESTIONS***

**1. Firstly, can you tell me more broadly about your organization: what it does, the people who work here, the people who access your services, and what your role is?**

Prompts if needed:

- Tell me about the health programs/services that you offer
- Who accesses your services? Are there any eligibility requirements?
- What about geographic reach – where do people live who access your services? Do you cover regional/rural/remote areas?
- Tell me about the staff and volunteers who work here (eg qualifications, roles)
- How are the service/programs funded?
- What is your role in the organization?

**2. Can you tell me about any role/s that your service currently plays in supporting your clients to access cervical screening - this might include anything from letting clients know about cervical screening (health promotion activities) to providing cervical screening tests, or supporting people to access tests and follow-up care?**

- **For those who play a role**
  - Can you step me through what this looks like in your service?
  - Who is involved? (Which staff play which roles)
  - What is the uptake/participation like for your clients in screening?
  - Prompt - are there any considerations for people living in regional/rural/remote areas?
- **For those who do not play a role at all**
  - Can you tell me why you don’t have a role?
  - Do you know how your clients access cervical screening? Tell me about this.
    - Prompt regarding rural/regional/remote
  - Do you know what % of your clients would access screening?
- **For both**
  - What about people in your focus population who live in regional or rural areas, are there additional challenges these people experience? Is there anything in place to support them?

**3. I’d like to ask you now more specifically about self-collection for cervical screening and what this option has meant, or could mean, for your clients and for your organisation**

- Before this interview, what was your level of knowledge and experience with self-collection?
- What do you think self-collection could mean/has meant for your clients?
  - Do you see any advantages or disadvantages?
- What has or does it mean for your organization?
  - (eg impacts or changes to the roles you can play, how you can support your clients)
- Does it offer new or different ways for you/your organisation to be involved in cervical screening?
  - How, tell me about this.
  - Why not, tell me about this.

**4. The National Cervical Screening Program guidelines provide some flexibility about the way self-collection is provided to people. I am interested in your thoughts or experiences with offering flexible models of cervical screening including self-collection. Examples may include telehealth, outreach and peer-led models but we are interested in other models you might have implemented yourself or know about.**

**Has your service implemented any flexible models of delivery?**

- **FOR THOSE WHO HAVE IMPLEMENTED FLEXIBLE MODELS**
  - Can you tell me about how this has worked in your service?
  - How did you get this happening?
  - What does/did this look like? (who is/was involved, when did you start?)
  - Did you overcome any challenges to implement this model? Tell me about this.
  - How have you managed the follow up for HPV positive clients?
  - What has the feedback from clients been like?
  - Have you developed any tools or resources as part of this that could be useful for others?
- **If you haven’t already implemented them,** **do you think any of these flexible models (telehealth, outreach, peer-led models) would work well in your service, for your clients?**
  - Why/why not?
- **Are there other alternative models that you are aware of that could work for your clients. These could be examples from other services that you offer for screening or testing that could be applied for cervical screening?**
  - Tell me about these
    - Prompts – are there any strategies have you used to implement any other models of service delivery that could be applied for cervical screening? Examples might include how pathology tests are managed, testing for other infections or shared care models

**5. I’d like to explore more about the main barriers and enablers that your organisation has experienced in providing or supporting access to cervical screening for your clients:**

- What would you say are the key things that have ***helped*** you/your service to support your clients to access cervical screening?
- On the other hand, is there anything that has made it ***harder*** for you to play a role in supporting your clients to access cervical screening? What would you say have been the main challenges?

**6. Thinking about the future, are there things that you, or your organization would need to make it easier (or possible) for you to improve the way you facilitate access to cervical screening for your clients?**

Prompts

- Infrastructure/equipment
- Support from your own organization
- Support from external organisations (eg pathology, clinical governance)
- Changes/clarity to guidelines/regulations/policy (eg more flexibility within the guidelines about when and where self-collection can be provided and by whom?)
- Additional resources/tools/training etc

**7. Additional questions about HPV Vaccination**
*Vaccination is a key pillar in the global strategy to eliminate cervical cancer as a public health issue (along with screening and access to early treatment), I am just going to ask you a few questions about vaccination now before we wrap up:*

| Does your service routinely provide immunisations, including catch up, to your clients?  *[read out response options]* | - Yes - all ages - Yes - children only - No but refer directly to practitioners/services who do when indicated - No this is not part of our services |
| --- | --- |
| ***If yes***, do you routinely provide HPV (also called Gardasil9) vaccine for catch-up or for older women? | - Yes or No - If no, why not? - If yes, how many doses do you offer? |
| Are you aware that from February 2023 only one dose of HPV vaccine is now needed up to age <26?  *[read out all options]*  Current schedule: One dose for EVERYONE <26 years (unless immunocompromised, then 3 doses). 3 doses for >26 years | - Yes and are giving one dose - Yes but not yet changed our practice - No was not previously aware |
| ***For those who are not currently offering HPV Vaccination:***  Given that only one dose of HPV vaccine is now needed and is funded under the NIP up to the age of 26 years, will this increase the likelihood of your service promoting or giving HPV vaccine? | - Yes - No - Not sure |
| What do you think are the implications of the change to one dose, or the extended catch-up age for your clients? | - Benefits? - Do you have any concerns about the impact of one dose eg if there is only one dose people may miss this altogether) and - what are you doing already, or do you think needs to be done to address the risk of people missing out? |
| Any additional comments/reflections about this: |  |

**Wrap up**

*Is there anything else you would like to add before we finish up?*

*Thank you for your time and for sharing your experiences and knowledge with us today*

*If you would like a copy of the transcript for this interview, we will email this out to you [ensure you have email address]*

*Are there any other people within the service [may have been mentioned earlier] that you suggest we speak to?*

*Can you provide their names and contact details now, or email these to [name]*

[Stop recording]

***Appendix – interview flow for interviewers***

***1. Demographics and background to service and interviewees***

***2. Explore the role that the service currently has in facilitating access to cervical screening for their clients***

*(roles, locations, models, systems, pathways etc)*

***3. Explore perceptions and experiences of self-collection for their organization and their clients***

*(level of knowledge and experience, perceived advantages, disadvantages and opportunities of self-collection, implications and opportunities for clients and for the organization)*

***4. Explore views and experiences of flexible models within the guidelines***

*What do they think about flexible models such as telehealth, outreach, peer-led.*

*Are they doing these? How do/would these work?*

*For those who have implemented flexible models, explore detail of implementation strategies*

***5. Explore Barriers and enablers to facilitating access to*** *Cervical Screening, including when implementing self-collection (perceived barriers if not currently implementing self-collection)*

***6. Explore what is needed to improve****/****enhance*** ***current cervical screening models*** *including for them to* ***start to provide self-collection*** *as part of service if they are not doing this already, or to improve implementation if they have started?*

***7. Explore roles and perceptions of one-dose HPV Vaccination and implications for their service***
